# Prevalence of gestational diabetes using IADPSG criteria in North India: A cross-sectional study

**DOI:** 10.6026/973206300214876

**Published:** 2025-12-15

**Authors:** Pranshi Asati, Swati Asati, Rahul Tiwari, Heena Dixit, Anil Managutti, Akriti Mahajan

**Affiliations:** 1 Department of Obstetrics and Gynaecology, Autonomous State Medical College, Lalitpur, Uttar Pradesh, India; 2 Department of Obstetrics and Gynaecology, Vyas Medical College & Hospital, Jodhpur, Rajasthan, India; 3 Department of Oral and Maxillofacial Surgery, Narsinhbhai Patel Dental College and Hospital, Sankalchand Patel University, Visnagar, Gujarat, India; 4 Department of Medical Health Administration, Index Institute, Malwanchal University, Index City, Nemawar Road, Indore, Madhya Pradesh, India; 5 Department of Oral medicine and radiology, Desh Bhagat Dental College and Hospital, Mandi Gobindgarh, Punjab, India

**Keywords:** Gestational diabetes mellitus, IADPSG criteria, prevalence, risk factors, pregnancy outcomes

## Abstract

Gestational diabetes mellitus (GDM) represents a growing challenge in India and is under-studied in many North Indian populations. In this cross-sectional research of 800 pregnant women at 24-28 weeks gestation in a tertiary hospital in North India, we found a GDM prevalence of 19.0% when applying IADPSG criteria. Key risk factors for GDM included maternal age over 30 years, pre-pregnancy BMI ≥25 kg/m^2^, family history of diabetes and prediabetes detected early in pregnancy. GDM was significantly associated with increased rates of caesarean delivery and neonatal macrosomia. The high prevalence underscores need for universal screening, earlier detection and tailored preventive measures in similar settings.

## Background:

Gestational diabetes mellitus (GDM) is defined as glucose intolerance first recognized during pregnancy and is associated with increased risk of hypertensive disorders, cesarean delivery, macrosomia, neonatal hypoglycaemia and long-term metabolic disease in both mother and child. India shows variable prevalence estimates because of differing diagnostic criteria, including DIPSI, WHO 1999, WHO 2013 and IADPSG. A recent pan-India meta-analysis estimated pooled GDM prevalence of ~13% (95% CI 9-16%) across multiple criteria, with higher estimates (≈17%) when using IADPSG/WHO 2013 in urban or North Indian settings. The same work shows that North India has pooled GDM prevalence ~16.1% (95% CI 9.9-22.7) from studies using various criteria [1]. A hospital-based research in South India applying IADPSG found age, BMI, thyroid disorders and heart disease as strong risk factors [2]. Another recent research in Kadapa (South India) showed that maternal age >35 years, pre-pregnancy BMI >26 kg/m^2^ and hypertension were significantly associated with GDM, along with psychosocial stressors [3]. Prediction models combining clinical and biochemical markers (fasting insulin, SHBG, HOMA-IR, TyG index) have shown good accuracy in South Indian populations when using IADPSG criteria [4]. The "Diabetes in Pregnancy Study Group of India (DIPSI)" versus IADPSG comparison in an Indian cohort showed that IADPSG criteria captured many more cases of GDM than DIPSI, highlighting that DIPSI misses a substantial fraction of women diagnosed under IADPSG [5]. Given these findings and that recent community-based data in Delhi show ~19.2% incidence of GDM using serial OGTTs and risk factors including older age, higher BMI and prediabetes [6], there remains a lack of hospital-based studies in North India exclusively using IADPSG with full risk-factor and outcome profiling. Therefore, it is of interest to report the prevalence of GDM using IADPSG criteria in a contemporary North Indian hospital cohort, its associated risk factors and obstetric outcomes.

## Materials and Methods:

This cross-sectional hospital-based research was carried out in a tertiary care centre in North India, between February and December 2024. Inclusion criteria were pregnant women aged 18-45 years, singleton pregnancy, at 24-28 weeks gestation, who had no pre-existing diabetes and who consented to participate. Women with multiple gestations or chronic conditions (e.g., steroid therapy, renal disease) were excluded. Based on expected prevalence ~17%, margin of error 3%, 95% confidence, sample size calculated was 780; accounting for non-response etc., 800 women were enrolled. Data on maternal age, parity, pre-pregnancy BMI (self-reported weight and measured height), family history of diabetes, early pregnancy glycaemic status (prediabetes if fasting plasma glucose in first trimester 92-125 mg/dL) and obstetric history were collected. All participants underwent a 75 g oral glucose tolerance test (OGTT) after overnight fast; venous plasma glucose measured at fasting, 1 hour and 2 hours. GDM diagnosis was made per IADPSG criteria (fasting ≥92 mg/dL, 1 h ≥180 mg/dL, 2 h ≥153 mg/dL). Obstetric outcomes recorded included mode of delivery, birth weight, macrosomia (birth weight ≥4.0 kg), neonatal hypoglycaemia, NICU admissions. Statistical analyses used chi-square test for categorical variables, t-test for continuous and multivariate logistic regression to derive adjusted odds ratios (aOR) with 95% confidence intervals. P-value <0.05 considered statistically significant.

## Results:

Out of 800 enrolled women, complete data were available for 770. Of these, 146 (19.0%) were diagnosed with GDM per IADPSG criteria. Analysis of maternal characteristics showed significant differences between women with and without GDM (Table 1 - see PDF). The mean maternal age among GDM women was 32.3 years compared to 28.1 years in the non-GDM group. More than half of the GDM group were overweight or obese, while only about one-third of non-GDM women had elevated BMI. Family history of diabetes was reported in 42.5% of GDM cases versus 14.9% in non-GDM. Early pregnancy prediabetes also differed significantly. Obstetric and neonatal outcomes revealed that GDM was associated with higher complications (Table 2 - see PDF). Caesarean section was performed in nearly 49% of GDM pregnancies compared to 30% of non-GDM pregnancies. Macrosomia occurred in 15.8% of neonates born to GDM mothers, more than double the frequency observed in the non-GDM group (6.7%). The mean birth weight was significantly higher in infants of GDM mothers (3.22 kg) compared with those of non-GDM mothers (2.95 kg).

## Discussion:

The observed prevalence of 19.0% GDM in this hospital-based North Indian cohort using IADPSG criteria is higher than many pooled national estimates (~13%) which combine various diagnostic criteria, but is consistent with higher urban/North Indian subsets reported in recent meta-analyses [7]. It aligns with findings from Delhi community-based cohorts showing ~19.2% incidence with age, BMI and prediabetes as key risk factors [8]. Risk factor profiling confirms that maternal age over 30 and elevated BMI are among the most consistent predictors of GDM in Indian settings [9, 10]. The role of early pregnancy glycaemic status (prediabetes) suggests that hyperglycaemia may begin before 24 weeks and supports argument for even earlier screening, similarly noted in the Kadapa research where pre-pregnancy BMI and hypertension were strong associations with GDM [8]. The family history of diabetes also emerges as a significant non-modifiable risk, indicating that genetic, environmental and possibly epigenetic factors may contribute. The obstetric outcomes demonstrate that GDM, when untreated or inadequately managed, increases risks of macrosomia and operative delivery. These results mirror findings from other Indian hospital-based observational studies, which show similar elevations in cesarean delivery and macrosomia rates in GDM cohorts diagnosed by IADPSG [11, 12]. These adverse outcomes underscore the clinical significance of even mild hyperglycaemia captured by IADPSG thresholds. Our research’s strength includes strict use of IADPSG criteria, adequate sample size and detailed risk factor and outcome data. However, limitations include hospital-based design, which may overestimate prevalence compared to community settings and potential recall bias of pre-pregnancy weight. Also, neonatal long-term outcomes and glycaemic control post diagnosis were not followed up. Comparison with other studies indicates considerable heterogeneity in prevalence based on criteria used; DIPSI often underdiagnoses relative to IADPSG [13, 14]. Prediction models combining biomarkers and clinical features may help in risk stratification to optimize resource use in constrained settings [15]. Implications for practice include advocating universal OGTT screening at 24-28 weeks using IADPSG criteria in similar North Indian tertiary centres, consideration for early screening among high risk (age >30, elevated BMI, family history, prediabetes) and implementing lifestyle interventions before and during pregnancy. For policy, there is a need to standardize diagnostic protocols across India to allow comparability and equitable care.

## Conclusion:

Approximately one in five pregnant women in this North Indian tertiary hospital were diagnosed with GDM using IADPSG criteria, with higher age, BMI, family history and early prediabetes as key risk factors. GDM was linked to higher rates of caesarean section and macrosomia. Future work should include population-based studies, early screening protocols and evaluation of interventions to reduce GDM incidence and improve maternal-neonatal outcomes.
